# Association of *ACE2* gene functional variants with gestational diabetes mellitus risk in a southern Chinese population

**DOI:** 10.3389/fendo.2022.1052906

**Published:** 2022-12-01

**Authors:** Gongchen Huang, Qiulian Liang, Yukun Wang, Linyuan Qin, Haili Yang, Lin Lin, Xiangyuan Yu

**Affiliations:** ^1^ Guangxi Key Laboratory of Environmental Exposomics and Entire Lifecycle Heath, Guangxi Health Commission Key Laboratory of Entire Lifecycle Health and Care, School of Public Health, Guilin Medical University, Guilin, China; ^2^ Scientific Experiment Center, Guilin Medical University, Guilin, China; ^3^ Laboratory of Gynecologic Oncology, Department of Gynecology, Fujian Maternity and Child Health Hospital, College of Clinical Medicine for Obstetrics and Gynecology and Pediatrics, Fujian Medical University, Fuzhou, China

**Keywords:** angiotensin-converting enzyme 2, gestational diabetes mellitus, risk, variant, association, functional

## Abstract

**Objective:**

To explore the relationship between angiotensin-converting enzyme 2 (ACE2) genetic variants and gestational diabetes mellitus (GDM) in a southern Chinese population.

**Methods:**

Potential functional variants (rs2106809, rs6632677, and rs2074192) of *ACE2* were selected and genotyped in 566 GDM patients and 710 normal pregnaõncies in Guilin, China. The odds ratio (OR) and its corresponding 95% confidence interval (CI) were used to evaluate the association between genetic variant and GDM risk, and then the false positive report probability, multifactor dimensional reduction (MDR), and bioinformatics tools were used to confirm the significant association in the study.

**Results:**

After adjusting for age and prepregnancy body mass index, logistic regression analysis showed that *ACE2* rs6632677 was significantly associated with a decreased risk of GDM (CC *vs*. GG: adjusted OR = 0.09, 95% CI: 0.01 – 0.71, *P* = .023; GC/CC *vs*. GG: adjusted OR = 0.68, 95% CI = 0.46 – 0.99, *P* = .048; and CC *vs*. GG/GC: adjusted OR = 0.09, 95% CI = 0.01 – 0.72, *P* = .024), whereas rs2074192 was associated with increased GDM risk (TT *vs*. CC/CT: adjusted OR = 1.38, 95% CI = 1.08 – 1.75, *P* = .009). Furthermore, we found that rs6632677 interacted with SBP (*P*_interaction_ = .043) and FPG (*P*_interaction_ = .021) and rs2074192 interacted with HDL-c (*P*_interaction_ = .029) and LDL-c (*P*_interaction_ = .035) to influence the GDM risk of the individual. In the MDR analysis, the rs6632677 was the best one-locus model, and the three-loci model was the best interaction model to predict GDM risk. In addition, functional analysis confirmed that rs2074192 may regulate the splicing process of *ACE2* gene.

**Conclusion:**

*ACE2* gene variants are significantly associated with the risk of GDM *via* gene–gene and gene–environment combinations. The rs2074192 C > T affects the splicing of the ACE2 gene, which may be a potential mechanism leading to the changed susceptibility of an individual female during pregnancy to GDM.

## Introduction

Gestational diabetes mellitus (GDM) is a complex disease caused by environmental and genetic factors, which are characterized by different degrees of impaired glucose tolerance during pregnancy. It is reported that the global prevalence rate of GDM is approximately 1%–14% (1). In China, the incidence rate of GDM is as high as 14.8% and is on the rise (2, 3). It is known that GDM can lead to serious maternal and infant complications, such as polyhydramnios, gestational hypertension, spontaneous abortion, pre-eclampsia (PE), preterm birth, respiratory distress syndrome, small for gestational age (SGA), large for gestational age (LGA), fetal macrosomia, shoulder dystocia, hypoglycemia, and even stillbirth (4, 5). In addition, GDM has a long-term impact on patients themselves and their offspring. For example, the risk of type 2 diabetes (T2DM) after childbirth is more than seven times higher than that of pregnant women with normal blood glucose, and the risk of metabolic diseases, such as obesity and T2DM, in their offspring is also greatly increased in the future (6).

It is generally believed that GDM has similar pathophysiological mechanisms as T2DM, such as insulin resistance and β-cell dysfunction. In addition, advanced gestational age, obesity, family history of T2DM, previous history of GDM, and previous poor obstetric history, etc., are also considered as risk factors for GDM (7–9). However, the specific etiological mechanism of GDM has not been fully revealed so far. Epidemiological evidence shows that GDM presents familial genetic characteristics. A family history of diabetes is an independent risk factor for GDM, and the closer the family relationship between diabetes patients and pregnant women, the greater the risk of GDM in pregnant women (6). Meanwhile, the incidence rate of GDM in Asian women during pregnancy is about three to seven times higher than that in Caucasians (10).

Single-nucleotide polymorphisms (SNPs) are DNA sequence variations caused by the conversion or transversion of a single nucleotide. Such genetic variants have the core information that determines the genetic susceptibility of human diseases and play important roles in the genetic anatomy of complex traits (11, 12). SNPs may act as quantitative trait loci (QTLs) and be associated with complex disease phenotypes by affecting the mRNA/protein levels, methylation rate, and physiological and biochemical indicators, etc. (13–15). For instance, studies show that the genetic variant rs10830963 of the *MTNR1B* gene is associated with the pathogenesis of T2DM by affecting the expression of *MTNR1B* (16), and the rs10830963G allele could significantly increase the odds of antenatal insulin therapy of GDM in pregnancy (17). We also found that angiotensin-converting enzyme 2 (ACE2) rs2106809 may be involved in the pathogenesis of PE as an expression quantitative trait locus (eQTL) that regulates the transcription of functional genes. These findings reveal that inherited genetic factors play a key role in the genesis and development of GDM.

ACE2, a key component of the renin angiotensin system (RAS), transforms angiotensin (ANG) II to Ang-(1–7) and protects against ANG II–induced oxidative stress and inflammation (18). It is reported that the ACE2 gene and its variants are closely related to T2DM risk or pregnancy complications such as fetal growth restriction (FGR), PE with placental insufficiency, SGA, oxidative stress (OS), and inflammation (19–22). Evidence suggests an association between ACE2 and glucose regulation. Ace2-knockout mice were more susceptible than wild-type mice to pancreatic β-cell dysfunction (23). ACE2 can inhibit the expression of insulin resistance (IR)–related cytokines and, thus, reverse IR (24). It is now known that placental ACE2 is significantly decreased from the first to the third trimester of pregnancy (25). Thus, we speculate that the decrease of ACE2 level during pregnancy may participate in the pathological development of GDM.

*ACE2* gene variants were significantly associated with multiple pregnancy complications and T2DM and its complications (19, 26–28). We propose the hypothesis that the functional genetic variations may be involved in the transcription or posttranscriptional regulation of *ACE2* and change the risk of individual GDM. Therefore, this study intends to clarify the contribution of genetic variants of the *ACE2* gene to GDM risk in a case-control study with 566 GDM patients and 710 normal pregnancies.

## Methods and materials

### Study population

**Figure 1:**
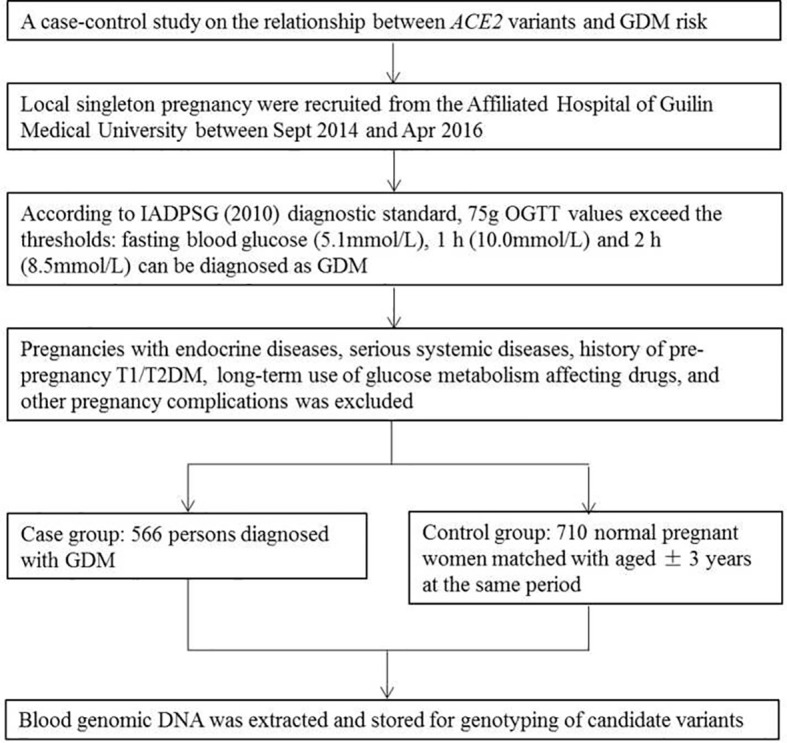
Study sample size screening flowchart.

All 1276 subjects (566 GDM cases with a mean age of 31.52 ± 4.72 years and 710 normal pregnancies ages 28.93 ± 4.23 years) were recruited from the Affiliated Hospital of Guilin Medical University between September 2014 and April 2016. Participants who met the diagnostic criteria for GDM recommended by the International Association of Diabetes and Pregnancy Study Group (IADPSG) in 2010 can be diagnosed as GDM if their 75 g oral glucose tolerance test (OGTT) values reach or exceed any of the following thresholds: fasting blood glucose (5.1 mmol/L), 1h blood glucose (10.0 mmol/L), and 2h blood glucose (8.5 mmol/L). The subjects need to have lived in Guilin for more than 2 years, have no kinship with each other, and have singleton pregnancies this time. If pregnancies had been previously diagnosed with endocrine diseases, serious systemic diseases, history of prepregnancy type 1 or type 2 diabetes, long-term use of glucose metabolism–affecting drugs, and other pregnancy complications, they were excluded. Each subject signed the informed consent, and the Ethics Committee of Guilin Medical University approved this research scheme. The screening flow of samples in this study is shown in **Figure 1**.

### Clinical and biochemical data

Clinical and biological characteristics were obtained from a unified questionnaire and patient medical records, including age, systolic blood pressure (SBP), diastolic blood pressure (DBP), fasting plasma glucose (FPG), triglyceride (TG), total cholesterol (TC), 75g OGTT 1h and 2h blood glucose, hemoglobin A1c (HbA1c), low-density lipoprotein (LDL), and high-density lipoprotein (HDL), etc. Meanwhile, the prepregnancy weight (Kg) and height (m) of the subjects were measured to calculate prepregnancy body mass index (pre-BMI) as weight (kg)/height (m)^2^.

### Genomic DNA extraction

Genomic DNA was extracted from EDTA-treated whole blood using a DNA extraction kit (Aidlab Biotechnologies Co.,Ltd, China) and stored at -80°C prior to PCR.

### Candidate variants selection

The potential functional variants in the *ACE2* gene region were screened by using the NCBI dbSNP database (http://www.ncbi.nlm.nih.gov/projects/SNP) and SNPinfo Web Server (http://snpinfo.niehs.nih.gov/). At first, 169 functional variants of the ACE2 gene region were identified by SNPinfo’s SNP function prediction tool with gene name as the keyword. Then, because the minimum allele frequency (MAF) of candidate variants is less than 0.05 in the Chinese Han Beijing population (CHB), 166 loci were excluded. Finally, three functional variants (rs6632677 G > C, rs2074192 C > T, and rs2106809 A > G) with linkage unbalance coefficient less than 0.8 between candidate variants were selected according to LD TAG SNP Selection of SNPinfo.

### Candidate variants genotyping by KASP method

**Figure 2:**
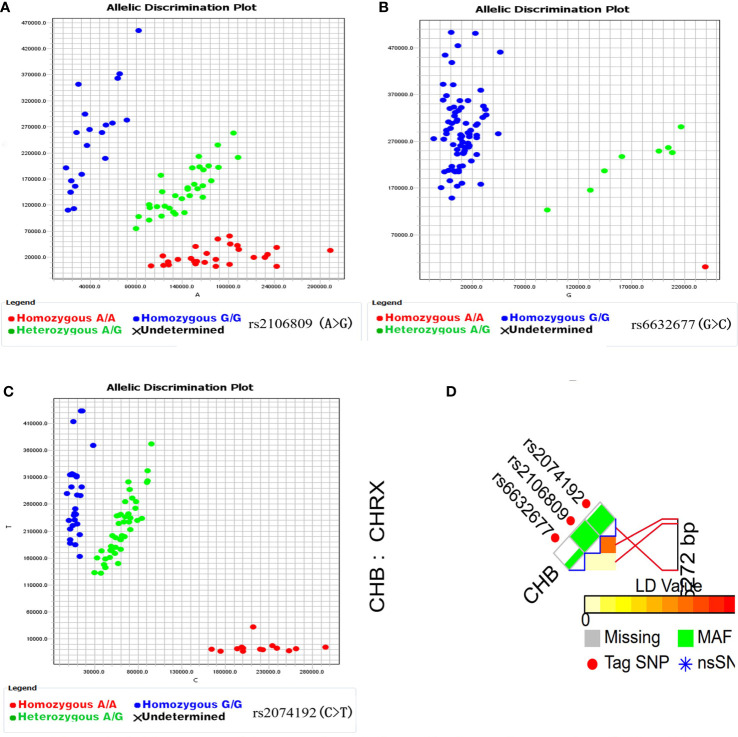
*ACE2* gene candidate genetic variations selection and genotyping (**A–C** are genotyping scatter plots of rs6632677 G>C, rs2074192 C>T, rs2106809 A>G respectively; **(D)** is SNP selection by SNPinfo Web Server online tool, LD: r^2^<0.8).

Candidate *ACE2* gene variants were genotyped by the competitive allele–specific PCR (KASP) method, and the corresponding specific PCR primers were designed and synthesized by Sangon Biotech (Shanghai) Co., Ltd. A volume of 10 µl reaction system was deposited in a 96-well plate, including 5 µl of FLu-Arms 2x PCR mix, 0.5 µl of 10 μM three specific primers (F1: 0.1µl, F2: 0.1µl, and R: 0.3 µl), 0.5 µl (25 – 150 ng) of DNA, and 4 µl of ddH_2_O. Two allele-specific forward primers were labeled with the fluorochrome FAM and HEX, respectively. Reactions were performed according to the following standard KASP-PCR program: predenaturation at 95°C for 3 min, then 10 touchdown cycles of 95°C for 15 s (denaturing), 61°C–55°C for 60 s (annealing and elongation), and followed by 30 cycles of 95°C for 15 s and 55°C for 60 s and plate read 30°C for 30 s. See **Figure 2** for the genotyping scatterplot of candidate loci.

### Statistical analysis

Statistical analysis was performed with IBM SPSS Statistics 28 for Windows (IBM Corp., Armonk, NY, USA). Clinical and biochemical variables are shown as mean ± SD ( $\bar{x}$ ± sd). The chi-square (χ^2^) test was adopted to detect the differences in selected demographic data. A χ^2^ goodness-of-fit test was performed to determine whether the distribution of genotypes of SNPs in the control group conformed to Hardy–Weinberg equilibrium (HWE). Binary logistic regression analyses were used to evaluate the associations between the genotypes of variants and the risk of GDM by calculating the odds ratios (ORs) and their 95% confidence intervals (CIs). In this study, the two-sided test was used, and *P* <.05 was considered statistically significant.

The false-positive reporting probability (FPRP) test described by Wacholder S et al. (29) was used to assess the robustness of the statistically significant associations detected in this study. During FPRP-value calculation, we first preset the prior probability of 0.1 and a relatively more stringent cutoff value of 0.2. Then, three values of odds ratios (OR = 1.2, 1.5, 2.0) that are most likely were set, assuming that there is a nonnull association. Finally, the OR estimate, 95% CI, and *P-*value associated with GDM risk of the genotype of the studied variant was entered to obtain the FPRP-value.

Multifactor dimension reduction (MDR) software (version 3.0.2) was applied to investigate the interaction effects between variants. Hundredfold cross-validation and 1000-fold permutation testing were adopted under the null hypothesis of no association, and the best multifactorial model was recognized with values of cross-validation consistency (CVC) and testing balanced accuracy (TBA) variables (30). In view of the fact that the research variation is located in the gene intron and may regulate gene transcription, the Alternative Splice Site Predictor (ASSP) tool was used to analyze their posttranscriptional splicing regulation (31). In the ASSP analysis, codon usage and stop codons for all three possible reading frames (F1 − frame 1, F2 − frame 2, F3 − frame 3) and scores of the preprocessing models reflecting splice site strength are calculated. We further applied GWAS4D online analysis (http://www.mulinlab.org/gwas4d/) (32) based on the GTEx database to analyze eQTL between studied variants and gene transcription levels.

## Results

### Characteristics of study subjects

The main characteristics of 566 GDM pregnancies and 710 healthy controls are shown in **Table 1**. Comparison of general data characteristics indicate that the age, BMI, SBP, DBP, TG, and HbA1c in GDM cases were significantly higher than those in the controls (*P* <.001). Also, the markers of glucose homeostasis, including FBG, 75g OGTT 1h blood glucose, and OGTT 2h blood glucose were also relatively higher (*P* <.001).

**Table 1:** Baseline characteristics of GDM patients and healthy pregnant controls (means ±SD).

	GDM (n=710)	Controls (n=567)	t	*P* value
Age (years)	31.52 ± 4.72	28.93 ± 4.23	-10.191	**<0.001**
Pre-BMI (kg/m2)	23.12 ± 3.61	21.59 ± 3.05	-8.044	**<0.001**
FBG (mmol/L)	5.23 ± 1.32	4.41 ± 0.36	-14.32	**<0.001**
OGTT 1h blood glucose (mmol/L)	9.77 ± 2.23	7.00 ± 1.43	-25.693	**<0.001**
OGTT 2h blood glucose (mmol/L)	8.30 ± 2.16	6.10 ± 1.10	-22.158	**<0.001**
SBP (mmHg)	111.47 ± 10.59	109.17 ± 9.36	-4.059	**<0.001**
DBP (mmHg)	70.42 ± 8.68	68.54 ± 8.02	-4.029	**<0.001**
HbA1c (%)	5.43 ± 0.69	4.95 ± 0.65	-12.901	**<0.001**
TG (mmol/L)	2.67 ± 1.21	2.39 ± 1.01	-4.505	**<0.001**
TC (mmol/L)	5.37 ± 1.16	5.27 ± 1.05	-1.687	0.088
HDL-c (mmol/L)	1.66 ± 0.42	1.65 ± 0.39	-0.333	0.739
LDL-c (mmol/L)	3.49 ± 1.03	3.40 ± 1.02	-1.512	0.131

BMI, body mass index; FBG, fasting blood glucose; SBP, systolic blood pressure; DBP, diastolic blood pressure; HbA1c, glycated hemoglobin; TG, Triglyceride; TC, Total cholesterol; HDL-c, high-density lipoprotein cholesterol; LDL-c, low-density lipoprotein cholesterol.

### Association between studied variants and the risk of GDM

#### *ACE2* rs6632677 G>C and GDM risk

Logistic regression analysis after adjustment of age and pre-BMI shows that *ACE2* rs6632677 was significantly associated with the risk of GDM. Compared with the GG genotype, the CC genotype can significantly reduce the GDM risk of individuals by 91% (CC *vs*. GG, adjusted OR = 0.09, 95% CI = 0.01 − 0.71, *P* = .023). Moreover, under the dominant model (GC/CC *vs*. GG) compared with the GG genotype, GC/CC genotype carriers have lower GDM risk (adjusted OR = 0.68, 95% CI = 0.46 − 0.99, *P* = .048). Under the recessive model (CC *vs*. GG/GC), the CC genotype carriers could significantly reduce GDM risk compared with GG/GC genotypes (adjusted OR = 0.09, 95% CI = 0.01 − 0.72, *P* = .024) as shown in **Table 2**.

**Table 2:** Association between genotypes of selected SNPs and GDM risk.

	Genotype	Case (n=566)	Control (n=710)	Crude OR (95%CI)^b^	*P* ^b^	Adjusted OR (95% CI) ^c^	*P* ^c^
rs6632677	GG	513 (90.6%)	621 (87.5%)	1		1	
	GC	52 (9.2%)	75 (10.5%)	0.84 (0.58-1.22)	0.357	0.79 (0.53-1.18)	0.254
	CC	1 (0.2%)	14 (2.0%)	**0.09 (0.01-0.66)**	**0.018**	**0.09 (0.01-0.71)**	**0.023**
	GC/CC	53 (9.4%)	89 (12.5%)	0.71 (0.49-1.02)	0.06	**0.68 (0.46-0.99)**	**0.048**
	GG/GC	565 (99.8%)	696 (98%)		1		1
	CC	1 (0.2%)	14 (2%)	**0.09 (0.01-0.67)**	**0.019**	**0.09 (0.01-0.72)**	**0.024**
rs2074192	CC	120 (21.4%)	143 (20.2%)	1		1	
	CT	199 (35.5%)	314 (44.4%)	0.76 (0.56-1.02)	0.067	**0.72 (0.52-0.99)**	**0.043**
	CT	199 (35.5%)	314 (44.4%)	0.76 (0.56-1.02)	0.067	**0.72 (0.52-0.99)**	**0.043**
	CT/TT	440 (78.6%)	564 (79.8%)	0.93 (0.71-1.22)	0.589	0.89 (0.67-1.19)	0.43
	CC/CT	319 (56.9%)	457 (64.6%)	1		1	
	TT	241 (43.1%)	250 (35.4%)	**1.38 (1.10-1.73)**	**0.005**	**1.38 (1.08-1.75)**	**0.009**
rs2106809	AA	155 (27.5%)	210 (29.6%)	1		1	
	AG	308 (54.3%)	361 (50.8%)	1.16 (0.89-1.50)	0.27	1.20 (0.91-1.57)	0.197
	GG	103 (18.2%)	139 (19.6%)	1.00 (0.72-1.40)	0.981	1.10 (0.78-1.56)	0.593
	AG/GG	411 (72.5%)	500 (70.4%)	1.11 (0.87-1.42)	0.389	1.17 (0.90-1.52)	0.235
	AG/AA	463 (81.8%)	571 (80.4%)	1		1	
	GG	103 (18.2%)	139 (19.6%)	0.91 (0.69-1.21)	0.532	0.98 (0.73-1.32)	0.884

Data presented as n (%), a Two-sided χ2 test for genotypes distributions between cases and controls, b Unconditional logistic regression analysis, c Adjusted for age, pre-BMI in logistic regression models.

Then, we utilized stratified analysis to evaluate the risk of rs6632677 C > G with GDM under a dominant genetic model. After adjusting for age and pre-BMI, compared with the GG genotype, GC/CC genotype carriers have lower GDM risk in the SBP (> 110.2 mmHg) subgroup (adjusted OR=0.45, 95% CI= 0.26-0.79, *P* = .005), in the FPG (≤ 4.77 mmol/L) subgroup (adjusted OR=0.46, 95% CI= 0.26-0.84, *P* = .021), and in the Hb (≤ 5.15 mg/dl) subgroup (adjusted OR=0.50, 95% CI = 0.25-0.99, *P* = .049). Interestingly, significant interaction effects between rs6632677 and SBP (*P*_interaction_ = .043) and FPG (*P*_interaction_ = .021) were detected, influencing individual’s susceptibility to GDM (**Table 3**).

**Table 3:** Stratification analysis for associations between ACE2 rs6632677 G>C and GDM risk.

Variables	GG (Case/Control)	CC/GC (Case/Control)	Crude OR (95%CI)	*P* ^a^	Adjusted OR (95%CI)	*P* ^b^	*P* ^c^
**Age (years)**							0.534
≤ 30.04	231 / 426	21 / 62	0.63 (0.37-1.05)	0.076	0.66 (0.38-1.12)	0.122	
> 30.04	282 / 195	31 / 27	0.79 (0.46-1.37)	0.409	0.73 (0.41-1.28)	0.727	
**Pre-BMI (kg/m2)**							0.416
≤ 22.3	232 / 410	21 / 61	0.61 (0.36-1.03)	0.062	0.62 (0.36-1.06)	0.078	
> 22.3	281 / 211	31 / 28	0.83 (0.48-1.43)	0.504	0.75 (0.43-1.32)	0.320	
**SBP (mmHg)**							**0.043**
≤ 110.2	245 / 359	29 / 42	1.01 (0.61-1.67)	0.963	0.96 (0.56-1.65)	0.892	
> 110.2	268 / 262	23 / 47	**0.48 (0.28-0.81)**	**0.006**	**0.45 (0.26-0.79)**	**0.005**	
**DBP (mmHg)**							0.409
≤ 69.2	252 / 334	29 / 47	0.82 (0.50-1.34)	0.422	0.77 (0.46-1.29)	0.314	
> 69.2	261 / 287	23 / 42	0.60 (0.35-1.03)	0.063	0.58 (0.32-1.03)	0.063	
**FPG (mmol/L)**							**0.021**
≤ 4.77	203 / 537	15 / 83	**0.48 (0.27-0.85)**	**0.012**	**0.46 (0.26-0.84)**	**0.011**	
> 4.77	310 / 84	37 / 6	1.67 (0.68-4.09)	0.261	1.74 (0.69-4.35)	0.239	
**Hb (mg/dl)**							0.372
≤ 5.15	136 / 399	11 / 59	0.55 (0.28-1.07)	0.079	**0.50 (0.25-0.99)**	**0.049**	
> 5.15	377 / 211	41 / 30	0.80 (0.49-1.32)	0.384	0.83 (0.49-1.40)	0.479	
**TG (mmol/L)**							0.960
≤ 2.55	273 / 405	26 / 55	0.70 (0.43-1.15)	0.157	0.68 (0.40-1.14)	0.139	
> 2.55	240 / 216	26 / 34	0.69 (0.40-1.18)	0.177	0.69 (0.38-1.23)	0.203	
**TC (mmol/L)**							0.515
≤ 5.30	251 / 342	25 / 43	0.79 (0.47-1.33)	0.379	0.73 (0.42-1.26)	0.258	
> 5.30	262 / 278	27 / 46	0.62 (0.38-1.03)	0.066	0.61 (0.36-1.06)	0.078	
**HDL-c (mmol/L)**							0.840
≤ 1.66	266 / 337	27 / 50	0.68 (0.42-1.12)	0.133	0.68 (0.40-1.17)	0.161	
> 1.66	247 / 284	25 / 39	0.74 (0.43-1.25)	0.259	0.68 (0.39-1.19)	0.173	
**LDL-c (mmol/L)**							0.828
≤ 3.41	262 / 339	24 / 46	0.68 (0.40-1.14)	0.138	0.62 (0.36-1.07)	0.088	
> 3.41	251 / 282	28 / 43	0.73 (0.44-1.21)	0.226	0.73 (0.42-1.26)	0.256	

a Unconditional logistic regression analysis,

b Adjusted for age, pre-BMI in logistic regression models,

c Test for multiplicative interaction obtained from logistic regression models.

#### *ACE2* rs2074192 C>T and GDM risk

It is shown that rs2074192 can significantly reduce the risk of individuals suffering from GDM; compared with the CC genotype, the CT genotype can significantly reduce the GDM risk by 28% (adjusted OR = 0.72, 95% CI = 0.52 − 0.99, *P* = .043). In addition, under the recessive model (TT *vs*. CC/CT), a significantly increased GDM risk of the TT genotype is detected compared with CC/CT genotypes (adjusted OR = 1.38, 95% CI = 1.08 − 1.75, *P* = .009) as shown in **Table 2**.

Stratified analysis reveals the significant associations between rs2074192 and age ≤ 30.04 years (adjusted OR = 1.41, 95% CI = 1.02 – 1.95, *P* = .036), the pre-BMI ≤ 22.3 kg/m^2^ group (adjusted OR = 1.40, 95% CI = 1.01 – 1.95, *P* = .042), the SBP ≤ 110.2 mmHg group (adjusted OR = 1.44, 95% CI = 1.03 – 2.01, *P* = .034), the DBP > 69.2 mmHg subgroup (adjusted OR = 1.43, 95% CI = 1.01 – 2.03, *P* = .044), TG > 2.55 mmol/L subjects (adjusted OR = 1.73, 95% CI = 1.19 − 2.54, *P* = .005), the TC > 5.30 mmol/L subgroup (adjusted OR = 1.71, 95% CI = 1.21 − 2.41, *P* = .003), HDL-c ≤ 1.66 mmol/L subjects (adjusted OR = 1.81, 95% CI = 1.29 − 2.54, *P* = .001), and LDL-c > 3.41 mmol/L subjects (adjusted OR = 1.82, 95% CI = 1.28 − 2.59, *P* = .001), respectively, under the recessive genetic model. Meanwhile, significant interaction effects between rs2074192 and HDL (*P*_interaction_ = .029) and LDL (*P*_interaction_ = .035) were detected. See **Table 4**.

**Table 4:** Stratification analysis for associations between ACE2 rs2074192 C>T and GDM risk.

Variables	CC/CT (Case/Control)	TT (Case/Control)	Crude OR (95%CI)	*P* ^a^	Adjusted OR (95%CI)	*P* ^b^	*P* ^c^
**Age (years)**							0.858
≤ 30.04	142 / 314	109 / 171	**1.41 (1.03-1.93)**	**0.031**	**1.41 (1.02-1.95)**	**0.036**	
> 30.04	177 / 143	132 / 79	1.35 (0.95-1.93)	0.098	1.34 (0.93-1.93)	0.112	
**Pre-BMI (kg/m2)**							0.713
≤ 22.3	202 / 376	161 / 196	**1.42 (1.04-1.95)**	**0.028**	**1.40 (1.01-1.95)**	**0.042**	
> 22.3	117 / 81	80 / 54	1.30 (0.92-1.84)	0.135	1.33 (0.93-1.90)	0.120	
**SBP (mmHg)**							0.625
≤ 110.2	158 / 266	114 / 133	**1.44 (1.05-1.98)**	**0.024**	**1.44 (1.03-2.01)**	**0.034**	
> 110.2	161 / 191	127 / 117	1.29 (0.93-1.79)	0.130	1.29 (0.91-1.82)	0.150	
**DBP (mmHg)**							0.893
≤ 69.2	166 / 252	114 / 128	1.35 (0.98-1.86)	0.064	1.32 (0.95-1.85)	0.098	
> 69.2	153 / 205	127 / 122	**1.40 (1.01-1.93)**	**0.045**	**1.43 (1.01-2.03)**	**0.044**	
**FPG (mmol/L)**							0.798
≤ 4.77	128 / 402	91 / 215	1.33 (0.97-1.82)	0.077	1.39 (1.00-1.94)	0.053	
> 4.77	191 / 55	150 / 35	1.23 (0.77-1.98)	0.385	1.19 (0.73-1.93)	0.489	
**Hb (mg/dl)**							0.451
≤ 5.15	84 / 302	63 / 153	**1.48 (1.01-2.17)**	**0.043**	**1.52 (1.03-2.24)**	**0.037**	
> 5.15	235 / 155	178 / 96	1.22 (0.89-1.69)	0.218	1.21 (0.86-1.68)	0.273	
**TG (mmol/L)**							0.078
≤ 2.55	176 / 288	122 / 171	1.17 (0.87-1.57)	0.309	1.19 (0.87-1.64)	0.280	
> 2.55	143 / 169	119 / 79	**1.78 (1.24-2.56)**	**0.002**	**1.73 (1.19-2.54)**	**0.005**	
**TC (mmol/L)**							0.135
≤ 5.30	166 / 247	106 / 137	1.15 (0.84-1.59)	0.390	1.09 (0.77-1.53)	0.672	
> 5.30	153 / 209	135 / 113	**1.63 (1.18-2.26)**	**0.003**	**1.71 (1.21-2.41)**	**0.003**	
**HDL-c (mmol/L)**							**0.029**
≤ 1.66	161 / 265	127 / 119	**1.76 (1.28-2.41)**	**0.001**	**1.81 (1.29-2.54)**	**0.001**	
> 1.66	158 / 192	114 / 131	1.06 (0.76-1.47)	0.738	1.03(0.73-1.45)	0.870	
**LDL-c (mmol/L)**							**0.035**
≤ 3.41	175 / 246	107 / 139	1.08 (0.79-1.49)	0.627	1.04 (0.75-1.46)	0.810	
> 3.41	144 / 211	134 / 111	**1.77 (1.27-2.46)**	**0.001**	**1.82 (1.28-2.59)**	**0.001**	

a Unconditional logistic regression analysis,

b Adjusted for age, pre-BMI in logistic regression models,

c Test for multiplicative interaction obtained from logistic regression models.

However, we failed to find significant associations between rs2106809 A>G and the risk of GDM in the present study (*P* >.05).

### False-positive reporting probability analysis

The FPRP test was adopted to assess the noteworthiness of the observed significant associations between the studied rs6632677 and rs2074192 and GDM risk. The prior probability of 0.1 and a relatively stringent FPRP cutoff value of 0.2 were set. The FPRP value was 0.155 for the association between rs2074192 and the risk of GDM under the recessive model (TT *vs*. CC/CT) and suggests that the significant correlation found above may be authentic. However, other significant associations found in the study may not be true, and the conclusion should be recognized with caution as shown in **Tables 5** and **6**.

**Table 5:** FPRP analysis for the significant associations of the rs6632677 G>C and GDM risk.

Comparisons	Adjusted OR (95%CI)	Prior probability					
		**0.25**	**0.1**	**0.01**	**0.001**	**0.0001**	**0.00001**
**rs6632677**							
CC vs. GG	0.09 (0.01-0.71)	0.693	0.871	0.987	0.999	1.000	1.000
GC/CC vs. GG	0.68 (0.46-0.99)	0.121	0.292	0.819	0.979	0.998	1.000
CC vs. GG/GC	0.09 (0.01-0.72)	0.697	0.874	0.987	0.999	1.000	1.000
**Subgroup**							1.000
SBP > 110.2 (mmHg)	0.45 (0.26-0.79)	0.236	0.481	0.911	0.990	0.999	1.000
FPG ≤ 4.77 (mmol/L)	0.46 (0.26-0.84)	0.258	0.510	0.920	0.991	0.999	1.000
Hb ≤ 5.15 (mg/dl)	0.50 (0.25-0.99)	0.319	0.584	0.939	0.994	0.999	1.000

Bold values indicate that the difference is statistically significant at the test level of α=0.2.

**Table 6:** FPRP analysis for the significant associations of the rs2074192 C>T and GDM risk.

Comparisons	Adjusted OR (95%CI)	Prior probability					
		**0.25**	**0.1**	**0.01**	**0.001**	**0.0001**	**0.00001**
**rs2074192**							
CT vs. CC	0.72 (0.52-0.99)	0.086	0.220	0.756	0.969	0.997	0.999
TT vs. CC/CT	1.38 (1.08-1.75)	0.058	**0.155**	0.669	0.953	0.995	1.000
**Subgroup**							
Age ≤ 30.04 (years)	1.41 (1.02-1.95)	0.088	0.225	0.762	0.970	0.997	1.000
Pre-BMI ≤ 22.3 (kg/m2)	1.40 (1.01-1.95)	0.092	0.233	0.769	0.971	0.997	1.000
SBP ≤ 110.2 (mmHg)	1.44 (1.03-2.01)	0.093	0.235	0.771	0.971	0.997	1.000
DBP > 69.2 (mmHg)	1.43 (1.01-2.03)	0.101	0.235	0.788	0.974	0.997	1.000
Hb ≤ 5.15 (mg/dl)	1.52 (1.03-2.24)	0.122	0.294	0.821	0.979	0.998	1.000
TG > 2.55 (mmol/L)	1.73 (1.19-2.54)	0.120	0.290	0.818	0.978	0.998	1.000
TC > 5.30 (mmol/L)	1.71 (1.21-2.41)	0.098	0.245	0.781	0.973	0.997	1.000
HDL-c ≤ 1.66 (mmol/L)	1.81 (1.29-2.54)	0.095	0.240	0.777	0.972	0.997	1.000
LDL-c > 3.41 (mmol/L)	1.82 (1.28-2.59)	0.102	0.255	0.790	0.974	0.997	1.000

Bold values indicate that the difference is statistically significant at the test level of α=0.2.

### High-order interaction with GDM risk by MDR analysis

MDR analysis indicates that rs6632677 made the best one-locus model for GDM with a maximum CVC of 100/100, a testing balanced accuracy of 0.5456, and a *P*-value of statistical test of.0009. Meanwhile, the three-loci model was the best interaction model to predict GDM risk with a maximum CVC of 100/100, a testing balanced accuracy of 0.5710, and a *P*-value <.0001 as shown in **Table 7**.

**Table 7:** MDR analysis for the GDM risk prediction.

Best model	Training balanced accuracy	Testing balanced accuracy	CVC	χ 2	*P*
1	0.5456	0.5456	100/100	10.93	0.0009
1,2	0.5689	0.5574	100/100	26.59	<0.0001
1,2,3	0.5773	0.5710	100/100	30.23	<0.0001

Labels: 1. rs6632677; 2. rs2074192; 3. rs2106809.

P-value for 1000-fold permutation test.

The best model was selected as the one in boldface with the maximum prediction precision and the cross-validation consistency (CVC).

### Potential regulatory function analysis

**Figure 3:**
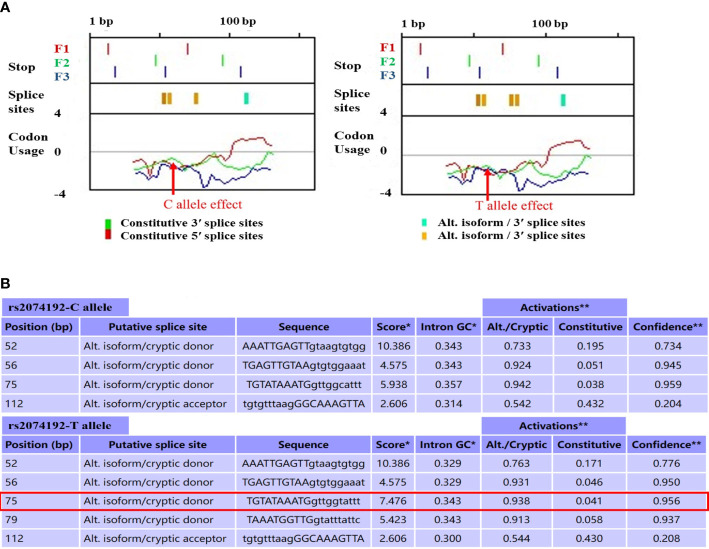
*ACE2* rs2074192 (C > T) potential regulatory function analysis by Alternative Splice Site Predictor (ASSP) **(A)** Schematic representation of splicing positions reflecting the *ACE2* rs2074192; **(B)** Score plot for preprocessing model reflecting sequences of putative splice sites; strenght: 161bp; Acceptor site cutoff: 2.2; Donor site cutoff: 4.5).

Alternative Splice Site Predictor (ASSP) tool analysis suggests that *ACE2* rs2074192 C>T may lead to changes in the activity of putative splice sites, which are characterized by score activation, intron GC activation, alt. isoform/cryptic activation, constitutive activation, and confidence activation near the polymorphism. For instance, from the analysis results, we noticed that the activity of the putative splice site in the 75 bp position of the examined DNA sequence had a score activation of 5.938, intron GC activation of 0.357, alt. isoform/cryptic activation of 0.942, constitutive activation of 0.038, and confidence activation of 0.959 with the C allele of rs2074192, whereas the rs2074192 T allele had a score activation of 7.476, intron GC activation of 0.343, alt. isoform/cryptic activation of 0.938, constitutive activation of 0.041, and confidence activation of 0.956. In addition, it seems that this mutation created a new splicing site at the 79 bp position of the examined DNA sequence only with the rs2074192 T allele but not the C allele as shown in **Figure 3**.

Furthermore, using the GTEx database, which incorporates 127 tissue/cell type–specific epigenome data sets, the GWAS4D online tool was used to analyze eQTL between rs2074192 and the regulation of gene transcription. This analysis indicated that rs2074192 could potentially regulate the expression of functional genes associated with GDM risk in a variety of specific tissues and cells, including *PIR* (total of 48 studies involved), and *VEGFD* (47 studies involved), which were identified as eQTL as shown in **Table 8**.

**Table 8:** The eQTL analysis on rs2074192 C > T and functional gene transcription levels by GWAS4D online tool.

rs#Position	RegulatedGene ID	No. of experimental studies (*P* < 0.05 / total)	Validated tissues and cell lines (*P* < 0.05)
chr X: 15582790	*PIR*	11 / 48	Adipose visceral omentum, Adrenal gland, Transformed fibroblasts, Colon transverse, Esophagus gastroesophageal junction, Esophagus mucosa, Esophagus muscularis, Nerve Tibial, Spleen, Testis, Thyroid
chr X: 15582790	*VEGFD*	14 / 47	Adrenal gland, Artery aorta, Artery tibial, Brain cortex, Transformed fibroblasts, Colon sigmoid, Esophagus mucosa, Heart atrial appendage, Liver, Lung, Muscle skeletal, Nerve Tibial, Testis, Thyroid

## Discussion

In this study, we propose the hypothesis that *ACE2* and its variants may participate in the pathological mechanism of GDM. Thereupon, a case control study involving 566 GDM patients and 710 normal pregnancies at the same time was conducted to investigate the relationship between *ACE2* functional variants and GDM. We found that rs6632677 G > C and rs2074192 C > T were significantly related to the incidence of GDM in Guilin women, and the mutant rs6632677 C allele and rs2074192 T allele showed an effect of reducing and increasing the risk of GDM, respectively. *ACE2* variants are likely to modify the genetic background and affect the risk of individuals suffering from GDM under the same environmental risk exposure. The findings of this study provide some genetic clues for the susceptibility of the Guilin population to GDM, and the specific mechanisms need to be further analyzed. Studies indicate that gene variants may be associated with disease susceptibility by gene–gene interaction or affecting complex traits, such as mRNA/protein/methylation levels, biochemical indicators, etc. (14, 15). Liu C et al. found that *ACE2* variants can significantly affect blood pressure (SBP or DBP) and blood lipid (TG, TC, HDL-C, or LDL-C) and regulate the onset of T2DM and its complications (33). This may be one of the mechanisms by which functional variants lead to individual susceptibility to disease. Therefore, we also carried out an association analysis between the studied variants and clinical traits of subjects.

Similar to this, we found that rs6632677 particularly affected the risk of GDM in SBP > 110.2 mmHg, FPG ≤ 4.77 mmol/L, and Hb ≤ 5.15 mg/dl subjects, and significant interaction effects between rs6632677 and SBP and FPG were detected. The associations between rs2074192 and GDM risk was even more pronounced in subjects of age ≤ 30.04 years, pre-BMI ≤ 22.3 kg/m^2^, SBP ≤ 110.2 mmHg, Hb ≤ 5.15mg/dl, TG > 2.55 mmol/L, HDL-c ≤ 1.66 mmol/L, and LDL-c > 3.41 mmol/L subgroups, and significant interaction effects were detected between rs2074192 and HDL-c and LDL-c. *ACE2* variants might alter an individual’s GDM risk by regulating the key physiological and biochemical variables of the organism. Meanwhile, although no significant association between rs2106809 and the risk of GDM was detected in the single variant analysis, a complex gene–gene combination was detected in the MDR analysis. That is, the three-loci model was the best interaction model to predict the risk of GDM. These findings indicate that *ACE2* gene variants show differences in effect among people with specific characteristics, and the combined effects or interactions between genetic and environmental factors (blood pressure, blood glucose, or blood lipid) may be one of the potential pathological mechanisms of GDM.

FPRP analysis is an effective method for determining the biological importance of associations (34). In this study, a strict FPRP threshold of 0.2 was set. In the recessive model, a significant correlation detected between rs2074192 and the risk of GDM risk was considered probably true and reliable. However, the FPRP values obtained in some comparisons that were much greater than 0.2 suggest that these associations may have been observed by chance. Therefore, the conclusions drawn here are preliminary and should be recognized with caution.

According to the research, genetic variants in the gene intron region can regulate mRNA transcription levels, thereby ultimately affecting an individual’s GDM risk (35, 36). In view of this, we explored the potential biological function of *ACE2*-positive associated variants using bioinformatics tools and found that rs2074192 is likely to regulate the posttranscriptional splicing process of *ACE2* by modifying the activity of splice sites with different alleles. The change of the rs2074192 allele from C to T can affect the efficacy of potential splicing sites and even directly erase an existing or create a new splice site. Furthermore, eQTL analyses using the GTEx database, which incorporates 127 tissue/cell type–specific epigenome data sets, suggest that rs2074192 could regulate the expression of various functional genes. This evidence may provide new research perspectives and clues for revealing the genetic mechanism of GDM susceptibility. However, it is still essential to explore the biological function of rs2106809 in future studies to verify its role in the pathogenesis of GDM.

This study explores the relationship between *ACE2* variants and GDM risk of Guilin, China, and finally, some etiological clues have been yielded from the perspective of genetics. However, this study still has some shortcomings. First, this was a hospital-based study and, therefore, might have selection bias. Second, potential influencing factors of GDM, such as smoking status, poor obstetrics, malnutrition, dangerous society, etc., were not measured, and these are likely to affect the final association effects between the studied *ACE2* functional variants and GDM risk. Third, although a large sample study design was adopted in this study, the very low frequency genotypes tested in studied variants may still limit the efficiency, especially in subgroup analysis. Fourth, this study did not experimentally explore the biological function of the significant association locus.

## Conclusion

*ACE2* genes rs6632677 and rs2074192 are significantly associated with the risk of GDM. The underlying mechanism may be that the single locus effects and/or complex gene–gene and gene–environment interactions regulate the transcription of *ACE2* gene and, thus, change the susceptibility of Guilin women to GDM during pregnancy.

## Data availability statement

The original contributions presented in the study are publicly available. This data can be found here: Dryad, Dataset, https://doi.org/10.5061/dryad.34tmpg4pb.

## Ethics statement

The studies involving human participants were reviewed and approved by Guilin Medical University and the Affiliated Hospital of Guilin Medical University. The patients/participants provided their written informed consent to participate in this study. Written informed consent was obtained from the individual(s) for the publication of any potentially identifiable images or data included in this article.

## Author contributions

XY and LL: protocol/project development and manuscript editing. GH, QL and YW: data collection and analysis, and manuscript writing. QL and LQ: data analysis and manuscript writing. HY: data collection and management. All authors contributed to the article and approved the submitted version.

## Funding

This study was supported by the Guangxi Natural Science Foundation of China (2020GXNSFAA23 8025); Guangxi Young and middle-aged teachers’ basic ability improvement project (2020KY12028); Maternal and Child Health Research Project of Guangxi Bagui Scholars (Zhang Jun); College Students’ Innovation Project (201810601031) of Guangxi, China; Fujian Provincial Health Technology Project (2020CXA017) and Fujian Provincial Maternity and Child Health Hospital Foundation (YCXM 20-16).

## Conflict of interest

The authors declare that the research was conducted in the absence of any commercial or financial relationships that could be construed as a potential conflict of interest.

## Publisher’s note

All claims expressed in this article are solely those of the authors and do not necessarily represent those of their affiliated organizations, or those of the publisher, the editors and the reviewers. Any product that may be evaluated in this article, or claim that may be made by its manufacturer, is not guaranteed or endorsed by the publisher.
